# Address in Surgery*Read before the Pennsylvania Medical Society, May 18, 1898

**Published:** 1898-11

**Authors:** W. L. Estes

**Affiliations:** South Bethlehem, Pa.


### ADDRESS IN SURGERY.*

BY W. L. ESTES, M.D., OF SOUTH BETHLEHEM, PA.

"Surgery has reached its zenith, and no advances of any value or magnitude may be expected in the future." This remark, said to have been made by a distinguished English surgeon some ten years ago, seems ludicrous indeed in reviewing the work done in surgery during the last year alone. This work is so considerable that I shall not, in the short time allotted to this address, attempt to give more than a sketch of a part of it, and then draw some conclusions which I trust may receive the endorsement of this society, the members of which are known to be thoughtful students of medical progress, and are accustomed to note the trend of scientific endeavors.

The brilliant operation of Schlatter, of Zurich, in successfully removing the whole stomach from a human being, stands perhaps foremost amongst the achievements of the year. As an exhibition of perfection in surgical technique, it is remarkable even in this day of asepsis; as a contribution to the physiology of digestion, it is even more valuable, however. While I do not believe it should set aside conclusively many deductions founded upon Dr. Beaumont's classicol experiments on the healthy stomach of Alexis St. Martin, it gives us most important suggestions with reference to the proper status of the stomach, and the unthought-of possibilities of intestinal digestion. The vicarious intestinal assumption of gastric function in Dr. Schlatter's patient was reached only after a long and very gradual process, as the history of the case shows.† The stomach of the old woman had, at the time of operation, been reduced to a small cavity with thick, indurated walls; every vestige of glandular tissue was choked by the cancer, so that its sole function at the time of operation and probably for some time previously, had been simply to serve as an imperfect passageway for the food into the intestines. What little digestion had been possible was done by the intestines; the stomach was not only the seat of the poison producing neoplasm, but was entirely cut out of the physiology of digestion.

Surgeons who wish to repeat Schlatter's operation should bear this in mind. They ought also to remember that Schlatter's case was unique in that while the whole stomach was involved, the neighboring glands and omentum were not implicated, and the stomach was still freely movable and unattached by adhesions. To find these conditions combined in the same patient must be exceedingly rare.

Another notable instance of ablation and the marvelous

---

*Read before the Pennsylvania Medical Society, May 18, 1898.

†See Medical Review, Dec 25, 1898.

adaptability of the gastro-intestinal tract was the case of Shepherd.* In extirpating a thirteen-pound fibromyoma of the mesentery, Shepherd was obliged by dense adhesions to remove seven feet eight inches of the small intestine of a man, who not only made a prompt recovery, but rapidly gained in weight afterwards.

Equally remarkable was the operation of Mr. Frederick Treves† in removing the whole descending colon and rectum from a child who had idiopathic dilatation of the colon. Colotomy having failed to relieve the child, about eight months after this operation Mr. Treves did the second operation, which consisted in exsecting the whole colon from its flexure in the left hypochondrium, and the rectum, and then bringing the end of the transverse colon down to the anus and suturing it there. The result was a complete relief of all the symptoms, and a happy recovery of the child.

Hepatic and renal surgery have had a great impetus during the last year. Mayo Robson, in England, Lange, Senn, Halsted, Murphy, and Fenger, in this country, have done excellent work in cholelithiasis; while the labors of Kelly, Weir, Fenger, Gerster and others have done much in advancing the technique of renal surgery. The impetus given by Kelly in this country, and by Pawlik on the continent, to ureteral catheterization, has led to routine examination of kidneys and ureters separately, and has made it possible to diagnose so surely that many brilliant operations on the ureters and kidneys have been performed. The future promises much for this line of work, and this will result in saving many kidneys and lives.

Besides this most excellent work Kelly has inaugurated, with the help of Noble, and a few other conservative men, an era of conservatism in gynæcology. These surgeons now enucleate uterine fibroids or myomata, without extirpating the organ itself; they recommend the enucleation of tumors of the broad ligament, without sacrificing the ovaries, and even treating puerperal septic metritis without sacrificing the uterus in many cases.

Kelly's demonstration of the possibility of the air-distension method for cystoscopic examinations of the bladders of men is also of great importance.

Dr. J. B. Murphy has shown that it is quite possible to unite divided blood vessels, and to repair torn vessels without obliterating their lumen or interrupting permanently the flow of blood through them, and he has thus made it possible to preserve important organs or members in case of accidental injury to their principal vessels.

The employment of skiagraphy, which promised much, has

---

*Montreal Medical Journal, December, 1897.

†Lancet, Jan. 29, 1898.

been shown to be of limited application, and while most useful in a restricted employment, the variability of tubes and currents, to say nothing of the plates used, make it very difficult to lay down any very helpful and certain rules for the general use of the X-rays. Besides, the very disagreeable burns which have resulted from too long exposures indicate that photographic investigations ought, in their mechanical parts, to be entrusted to a limited number of experts, with fixed, properly tested, and well-known apparatus. The fluoroscope, however, is a most valuable agent in employing X-rays, and even to unskillful operators, with a little practice, it will render fruitful results.

The addition of formaldehyde to the sterilizing armamentarium has proved a valuable acquisition. For sterilizing knives and edged instruments generally (by its vapor), it seems almost an ideal agent; and it will perhaps soon divide honors with dry heat and steam for the sterilization of dressings.

The recrudescence of the very important discussion of how best to sterilize and prepare the hands for aseptic operations has resulted in the adoption very generally of some form of glove. The ideal glove material has yet to be discovered, but thin rubber gloves seem to have the best endorsement at present.

Schleich's general anæsthetic mixture, which at first seemed to promise much, has not received the hearty endorsement of American surgeons. In an experience of thirty-eight cases, the writer has found that while anæsthesia is easier as a rule, and cyanosis is not as common as with ether, vomiting, especially late vomiting, is just as common and more distressing. Dr. Willy Meyer, in a recent letter published in the *Medical Record* (*Medical Record*, April 22, 1898), called attention to some investigations made by Dr. Weidig at his (Dr. Meyer's) suggestion, which showed that the ordinary Schleich's mixtures are really mixtures, and not genuine solutions. The chloroform, petrolic ether, and sulphuric ether partly combine; no free chloroform could be found, but in each of the three solutions free sulphuric ether in varying proportions was found. Dr. Meyer also mentions the fact that Meltzer, who investigated the physiological effect of pure petrolic ether for him, found that it acts as a tetanizing agent on animals. It may prove not to be the innocent diluent that Schleich believed it was. The mixture should therefore be used with great caution.

Traumatic surgery has received scant consideration during the last year; indeed for many years surgical investigations have been confined almost wholly to pathologic surgery. The distinctions between "general surgery" and "gynecologic surgery" will possibly soon disappear, and the two be merged, but there is gradually developing another special branch of surgery, which may be called traumatic surgery. In times of peace this specialty will belong chieflv to surgeons connected with railway, mine, and mill hospitals. It is a great pity that this class ot surgeons, on account of excessive work and modesty, has not given the profession the product of its labors and experience in well-considered, systematic, and comprehensive monographs. The present "little unpleasantness" with Spain may serve to give a fresh impetus to acute surgery, and in the next few years traumatic surgery may again rank amongst the foremost endeavors of our progressive age.

It is a great mistake to believe that the "last word has been said" in traumatic surgery. Antisepsis and asepsis have done marvels for conservative attempts, and in lowering the general death rate after injuries. Of very great importance, in this branch of surgery, is the fact that acute anæmia is the dominant factor in ordinary surgical shocks. The writer, in urging this doctrine about ten years ago, gave certain comparative statistics from his own work to emphasize his point. In 1894 in a monograph entitled "A Contribution to the Study of Modern Amputations,"* this doctrine was again brought forward, and it was shown that by practice founded upon it, the mortality rate in a series of 180 single major amputations (which included seven hip-joint amputations, with only one death) was reduced to 2.77 per cent. against his former rate of 7.89 per cent. in 114 cases; the double synchronous amputations in 25 cases was reduced to 12 per cent. mortality, against 46.25 per cent. in 13 cases, after the old practice. Since the publication of the foregoing statistics, there have been 84 single major amputations and 2 deaths, a mortality rate of 2.38 per cent., and 17 multiple amputations and 2 deaths—11.7 per cent. mortality. In writing some years ago on the mortality rate of modern amputations, the writer stated that he believed the results after single major amputations ought to be as good as those after the average ovariotomy operations. It seems he has now the satisfaction of having realized this anticipation. The argument for the method—if one can call it a method—is, as observation shows, that by far the majority of deaths after modern amputations occur within forty-eight hours after operation, from exhaustion or shock, and as this so-called shock is a condition brought about by acute anæmia (great loss of blood), it is bad surgery to operate upon a person reduced almost to a moribund condition, when by careful antisepsis and the employment of elastic constriction over the crushed tissues, no evil is likely to result for thirty or forty hours, and during this time by the employment of active stimulation with strychnine and saline enemata

*Medical Record, Nov. 3. 1894.

the patient may again have his vessels filled with blood, and be brought to a condition which will bear a major operation.

The subject in acute or traumatic surgery which has received some commensurate consideration during the last year is fractures. This renewed discussion is probably due to the interest given the subject by the employment of X-rays, and the comparative accuracy with which the friends of the patient, as well as the surgeon, may now determine how well the bone has been "set." In this field the writer predicts that radiographs are likely to be disturbing factors as well as useful instruments, and that they will multiply suits for malpractice and damages until a long series of investigations and observations shall prove that it is the exception and not the rule for the surgeon to be able to make exact coaptation and retention of the bony fragments after a so-called simple fracture. The general tendency of to-day is the simplification of apparatus, and the allowance of more freedom of motion and locomotion in the treatment of fractures. Of far greater importance, however, is the gradually growing belief that the majority of so-called "simple fractures" are far from being so indeed, and that explorations by incision and adjustments of fragments by sight are not only justifiable, but in many instances absolutely essential for proper treatment. An accumulating array of instances has shown these incisions do not augment the danger when the operation is done with proper aseptic precautions, and that they markedly improve the results of treatment. The open method of treating fractures in proper cases is now not only the safe method, but the proper and imperative method of handling many fractures.

The most opportune and most suggestive paper of the year was the one read before the Practitioners' Society of New York, by Dr. McBurney, on "When to Operate." This is a vital question in surgery, and one which in many cases is far from settled. This is also a question in which surgeons ought to strive to indoctrinate family practitioners, for upon them in many instances this matter bears most heavily. Many organs and members have been sacrificed, and many lives lost because the medical man in charge had not made up his mind, or did not know when to operate. The discussion of Mr. Marmaduke Shields' paper on "Immunity and Latency After Operations for Cancer of the Breast,"* before the Royal Medical and Chirurgical Society of England, by such men as Pearce Gould, Watson Cheyne, W. H. Bennet and Fred Treves, shows that the whole question of cancerous invasion, growth, and cure are far from settled, even in the most maturely thoughtful and progressive minds. It is generally conceded now-a-days, however, that preceding what may be called the invasion

---

*Lancet, Feb. 26, 1898.

of cancer, there is a pre-cancerous stage and that cure may be confidently expected if the tumor be thoroughly removed during this first stage. This fact ought to be thoroughly established, and be received by the whole profession as a matter of the most vital importance. Family physicians should be made to believe that an incipient tumor should at once be submitted to the judgment of a surgeon, and that it is criminal to allow a patient to wait until ulceration or enlarged lymphatic glands disclose the growth to be malignant. These pathognomonic signs also declare in many instances, the death sentence of the unfortunate patient; they indicate, also, that an extensive operation must be done, and the chances of recurrence at this time are greater than those of thorough eradication.

The removal of benign tumors ought also to be done as early as possible. Constant irritation, growth under pressure, or some dyscrasia of the patient operate to make simple papillomata; fibroids and adenomata take on sarcomatous (myxomatous) degenerations, and the borderlands are so close that it is rarely ever safe to permit these tumors to develop as they will. It is far simpler, easier, and safer to remove these tumors in their very early growth, and it should be done.

The danger of general tubercular infection from a single focus of invasion is too little appreciated by physicians. The persistent enlargement of lymphatic glands, especially about the necks of children, those of diathetic parents especially, should receive early attention, and the knife ought to be used before they break down and suppurate. Inflammations involving the bones of diathetic children, especially a condition suggesting an osteomyelitis, require early radical operations. Many bones and limbs may thus be saved.

Now-a-days intestinal obstruction from any cause, but especially the so-called mechanical ones and those from hernia, should not be allowed to go on until a "typhoid" condition and fecal vomiting indicate that profound septicæmia and gangrene of the gut have taken place. Dr. Fred Krammerer, of New York, has called attention recently to a sign which nearly every surgeon of much experience has noticed, namely, circumscribed peristalsis, which if apparent to the sight at a very much distended point of the abdomen, is indicative of complete obstruction. When this sign is observed with the other ordinary signs and symptoms of obstruction, no time should be lost in performing an operation.

One would suppose that the much-written subject appendicitis had been so thoroughly discussed that little remains to be said on the matter of when to operate, and yet I dare say it is the experience of nearly every surgeon in active practice to see cardinal rules neglected nearly every week. It is so well established that unless a case of appendicitis improves in forty-eight hours an operation is imperative, one is astonished to find so many cases allowed to suffer, and in many instances to die, because this rule is neglected or not known. Furthermore, the very important rule that an operation between attacks ought to be performed after a recurring attack of appendicitis of even a mild (so-called catarrhal) type, is entirely too often neglected.

Recent work in renal surgery has shown the great good of early operative exploration, and removal of calculi from the pelvis of the kidney. As soon as persistent and recurring pain, and microscopic and chemic examination of the urine, obtained separately by ureteral catherization, have determined the probable presence of a stone in the kidney, an early removal of the stone will save the organ and restore the patient to health. It is of vital importance to remove a tubercular kidney as soon as the diagnosis can be made, and the other kidney determined to be healthy.

Later teaching and clinical experience show the great importance of early operation in obstruction of the bile ducts—especially the common duct. To wait for persistent jaundice with frequent rigors, high temperatures and frequent vomiting, before determining to operate, exposes the patient to fatal septicæmia, and complicates the operation by inflammatory adhesions, besides the great danger of perforation and general septic peritonitis.

I might continue these instances beyond your patience to hear. I trust I have said enough, however, to suggest to you the very great importance of recognizing the proper time for operating, and how vital it is in many instances not only to know this, but to faithfully and firmly practice it. There are a great many conditions, unfortunately, which remain still in doubt. To the study of these, surgeons should now give more attention. In order to know when to operate in any given case, it is necessary to fully understand and to appreciate the pathology of the disease or traumatism, as well as its clinical history; this feature of surgical investigation is too much neglected, especially in our country.

A recent notice of Stephen Paget's book on John Hunter, which appeared in the *Philadelphia Medical Journal*,* very aptly sums up what seems to me the lesson to be learned from a review of the year's work in surgery. It says: "The lesson of Hunter's life was never more needed than to-day, when mechanical conceptions of surgery are so dominant, and when technic has so largely replaced pathology." Improved technique has unquestionably greatly widened the bounds of

*Philadelphia Medical Journal, March 26, 1898.

surgery, and opened up vistas which to the daring and pushing surgeon seem well-nigh illimitable. The field of the general surgeon is encompassing that formerly sacred to the gynæcologist, and now encroaches upon the domain formerly given over wholly to the physician. Are we not going a little too fast? Empirical precedents and undigested observation and experience do not furnish stable ground-work for lasting scientific facts. It seems to me we are in grave danger of outrunning physiologic and pathologic investigations in many surgical attempts. It is time "to cast an anchor to the windward" and wait for proper observations and the careful working out of our present location and bearings. Surgeons, of the United States especially, very much need more laboratories and fewer clinics.

Besides the prevalence of "mechanical conceptions of surgery," another serious matter is that the furor for operating and the lust for extirpations have taken fast hold upon us. The surgeon who can not enumerate his cases of organs removed by the scores is a beginner indeed, and has but reached the stage where he must rush into print with a new device or "an improved method" for a pusher on the high road leading to his century count! Radicalism has been rampant; it is high time painstaking, thoughtful and scientific conservatism should have a fair chance.—*Pennsylvania Medical Journal.*

